# Predictors and trajectories of antibiotic consumption in 22 EU countries: Findings from a time series analysis (2000–2014)

**DOI:** 10.1371/journal.pone.0199436

**Published:** 2018-06-22

**Authors:** Maria Michela Gianino, Jacopo Lenzi, Marco Bonaudo, Maria Pia Fantini, Walter Ricciardi, Gianfranco Damiani

**Affiliations:** 1 Department of Public Health Sciences and Pediatrics, Università di Torino, Turin, Italy; 2 Department of Biomedical and Neuromotor Sciences, Alma Mater Studiorum—Università di Bologna, Italy; 3 Department of Public Health, Università Cattolica del Sacro Cuore, Fondazione Policlinico ‘Agostino Gemelli’ IRCCS, Rome, Italy; National Institute of Health, ITALY

## Abstract

**Background:**

This study analyzes the trajectories of antibiotic consumption using different indicators of patients’ socioeconomic status, category and age-group of physicians.

**Methods:**

This study uses a pooled, cross-sectional, time series analysis. The data focus on 22 European countries from 2000 to 2014 and were obtained from the European Center for Disease and Control, Organization for Economic Co-operation and Development, Eurostat and Global Economic Monitor.

**Results:**

There are large variations in community and hospital use of antibiotics in European countries, and the consumption of antibiotics has remained stable over the years. This applies to the community (b = 0.07, p = 0.267, 95% -0.06, 0.19, b-squared <0.01, p = 0.813, 95% = -0.01, 0.02) as well as the hospital sector (b = -0.02; p = 0.450; CI 95% = -0.06, 0.03; b-squared <0.01; p = 0.396; CI95% = > -0.01, <0.01). Some socioeconomic variables, such as level of education, income, Gini index and unemployment, are not related to the rate of antibiotic use. The age-group of physicians and general practitioners is associated with the use of antibiotics in the hospital. An increase in the proportion of young doctors (<45 years old) leads to a significant increase in antibiotics consumption, and as the percentage of generalist practitioners increases, there use of antibiotics in hospitals decreases by 0.04 DDD/1000 inhabitants.

**Conclusions:**

Understanding that age-groups and categories (general/specialist practitioners) of physicians may predict antibiotic consumption is potentially useful in defining more effective health care policies to reduce the inappropriate antibiotic use while promoting rational use.

## Introduction

Antibiotic resistance is a major public-health problem of global importance because it is related to treatment failure, increased use of health care services and increased mortality [1,2]. Consumption and over-consumption of antibiotics are recognized as the main cause of antibiotic resistance.

In response to this problem, the European Union put in place over time community strategies and action plans supporting, as pillar, antimicrobial stewardship, defined as a coherent set of actions which promote using antimicrobials responsibly. Antimicrobial stewardship aimed to provide evidence-based data on possible links between consumption of antimicrobial agents and the occurrence of antimicrobial resistance in humans and food-producing animals and, then, to develop EU guidelines for the prudent use of antimicrobials in human medicine and to assist Member States implement EU guidelines for the prudent use of antimicrobials in veterinary medicine [3]. At national level in Europe, many countries have implemented antibiotic stewardship programmes, at national or regional level. These initiatives provide for local surveillance of antibiotic consumption, systematic measuring, evaluating & improving quality of antibiotic usage, regular training of prescribing physicians and other relevant healthcare workers in diagnostics, treatment and prophylaxis of infections, focusing on appropriate use of antimicrobial agents as well as prevention and control of antimicrobial resistance. For example some antibiotic stewardship strategies in European countries are based on educational resources (UK and Germany), on public reporting with the data on antibiotic consumption and resistance for hospital and primary care publicly available on a website (UK) or on cross-sectoral antibiotic stewardship networks implemented in different settings (hospital, primary care, long-term care facilities) and at local, regional and national levels (Sweden France, Spain) [4,5]. Reliable and comparable data on the patterns of national antibiotic drug use and distribution are the starting point for analyzing the antibiotic resistance problem.

Since late 2005, the understanding and the measurement of inequalities in health and health-care use has been identified as a priority by the WHO Commission on Social Determinants of Health[6]. Several studies have investigated the role of socioeconomic determinants in facilitating inequalities in health and health-care use. Most of the previous studies that assessed the impact of socioeconomic determinants on antibiotic consumption have focused on different countries and time-points[7,8]. The few ones analyzing the role of socioeconomic determinants on antibiotic consumption at the European level have focused on different settings, such as the hospital or outside the hospital [9,10].

Several studies measure particular aspects of socioeconomic parameters, such as educational level, rather than investigate a set of two or more determinants.

This study analyzes the trajectories of antibiotic use across 22 EU countries and it assesses: (i) how the antibiotic consumption has changed in the community and hospital sectors over a 15-year period; (ii) the correlations between antibiotic use and a variety of socioeconomic determinants; (iii) the correlations between antibiotic use and categories of prescribing physicians or age of physician, which is used as a proxy for experience, and the background of the prescribing physician.

## Methods

This study used a pooled cross-sectional time series analysis of secondary data for 22 European countries between 2000 to 2014. These countries and years were chosen based on the availability of data. The unit of analysis was each country in each year (country-year). The countries included in the study were the following: Austria, Belgium, Czech Republic, Denmark, Estonia, Finland, France, Germany, Hungary, Iceland, Ireland, Italy, Latvia, Lithuania, Luxembourg, Netherlands, Norway, Slovakia, Slovenia, Spain, Sweden and the United Kingdom.

Official data were obtained from the European Center for Disease and Control (ECDC), Organization for Economic Co-operation and Development (OECD), Eurostat and Global Economic Monitor (GEM). The indicators considered are related to: the consumption of antibiotics in two different sectors, that are primary care and hospital; the prescribing physicians stratified in five age groups, which are 35–44, 45–54, 55–64, 65–74; the physician categories, that are generalist, specialist and not further defined medical doctors; and related to socio-economic determinants such as the GINI coefficient, household income and education level of the population aged 25–64, stratified into three classes.

These indicators are shown in Table 1, which illustrates the definition and source for each of them.

**Table 1:** Variables, indicators, definitions and data sources.

	Indicators	Definition	Source
**1**	Consumption of Antibacterials for Systemic Use (ACT group J01) in primary care sector	DDD per 1000 inhabitants and per day	ECDC 2017
**2**	Consumption of Antibacterials for Systemic Use (ACT group J01) hospital sector	DDD^α^ per 1000 inhabitants and per day in	ECDC 2017
**3**	Gini Coefficient of equivalised disposable income	Gini coefficient^β^ (scale 0 to 100)	EUROSTAT 2017
**4**	Income of households^γ^ –Disposable income	Euro per inhabitant	EUROSTAT 2017
**5**	Educational attainment level^δ^ (0–2)—population 25–64 years	Less than primary, primary and lower secondary education (%)	EUROSTAT 2017
**6**	Educational attainment level^δ^ (3 and 4)—population 25–64 years	Upper secondary and post-secondary non-tertiary education (%)	EUROSTAT 2017
**7**	Educational attainment level^δ^ (5–8)—population 25–64 years	Tertiary education (%)	EUROSTAT 2017
**8**	Unemployment	Total (% of total labor force) (modeled ILO estimate)	GLOBAL ECONOMIC MONITOR 2017
**9**	Physicians—under 35 years old	% of total physicians (head counts)	OECD 2017
**10**	Physicians—35–44 years old	% of total physicians (head counts)	OECD 2017
**11**	Physicians—45–54 years old	% of total physicians (head counts)	OECD 2017
**12**	Physicians—55–64 years old	% of total physicians (head counts)	OECD 2017
**13**	Physicians—65–74 years old	% of total physicians (head counts)	OECD 2017
**14**	Physicians—Generalist medical practitioners	% of total physicians (head counts)	OECD 2017
**15**	Physicians—Specialist medical practitioners	% of total physicians (head counts)	OECD 2017
**16**	Physicians—Medical doctors not further defined	% of total physicians (head counts)	OECD 2017

Abbreviations: OECD, Organization for Economic Co-operation and Development; ECDC, European Center of Disease and Control; DDD, Defined Daily Dose.

α: The DDD is the assumed average maintenance dose per day for a drug used for its main indication in adults. This indicator is used to assess antibiotic use in the hospital sector and the community and it is an internationally accepted parameter for making comparisons between countries

β: Gini Coefficient: the Gini coefficient measures the extent to which the distribution of income within a country deviates from a perfectly equal distribution. The perfect equality is expressed with 0 and full inequality is expressed with 100.

γ: total disposable income of a household is calculated by adding together the personal income received by all of household members plus income received at household level; that includes: all income from work (employee wages and self-employment earnings); private income from investment and property; transfers between households; all social transfers received in cash including old-age pensions.

δ: the level of educational attainment is defined according to the International standard classification of education (ISCED). The educational attainment level of an individual is the highest ISCED level successfully completed. Educational attainment levels are usually presented for three main categories: i) less than primary, primary and lower secondary education (ISCED 2011 levels 0–2); ii) upper secondary and post-secondary non-tertiary education (ISCED 2011 levels 3 and 4); iii) tertiary education (ISCED 2011 levels 5–8).

### Statistical analyses

To assess the relationship between dependent and independent variables over the 15-year study period, the study adopted a pooled, cross-sectional, time series design. Specifically, this design involved observing the variables for different cross-sections over a given timespan [11]. The dependent variables were indicators 1 and 2, while the independent variables were indicators 3 to 16.

A fixed-effects linear regression was done by performing a Hausman test with Sigmamore option [12] because the random-effects specification was found to be inappropriate for country-level effects in the adopted model. One advantage of fixed-effects models is that they control for time-invariant heterogeneity among countries [13]. The presence of exogenous time trends in both the dependent and independent variables (i.e., time-fixed effects) was controlled by adding dummy variables to the model for each of the study years except the first.

To avoid model overfitting, indicators 3 to 16 were halved by collapsing age groups, while secondary and tertiary education levels (6 and 7) were merged because they gave similar results. Additionally, no results were reported for indicators 9 and 16 because they complement the 100 indicators shown in the Results section. The relationship between all the remaining dependent and independent variables were separately examined, resulting in 12 distinct fixed-effects models. This choice was driven primarily by concerns about model over-fitting and multicollinearity.

For all the analyses, the significance level was set at *p* < 0.05, and listwise deletion was used. The significance of each independent variable was assessed using robust standard errors due to results obtained from performing a modified Wald test for group-wise heteroskedasticity in the regression residuals [14]. All data sets were analyzed using the Stata software package, version 13 (StataCorp. 2013, Stata Statistical Software: Release 13; StataCorp LP, College Station, TX, USA).

## Results

**Fig 1:**
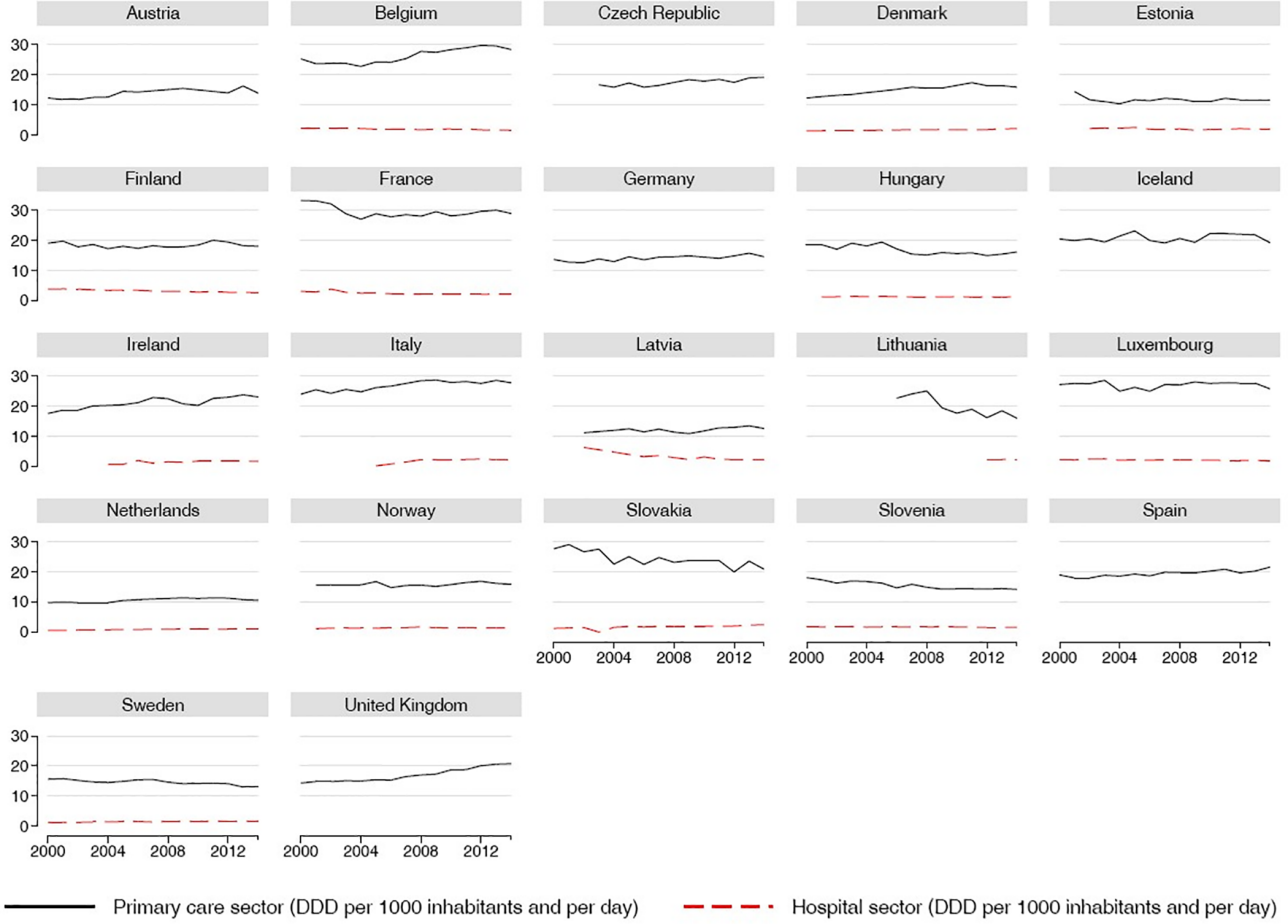
Antibiotics use pattern.

The antibiotic use patterns in hospital and community settings is shown in Fig 1.

The figure reveals considerable variability among the countries regarding the consumption in primary care sector in 2014. The consumption pattern in the Netherlands was approximately 10 DDD, while in Belgium, France, Italy and Luxembourg, the pattern was approximately 30 DDD at the end of the observation period. Five countries (Belgium, France, Italy, Luxembourg, and Slovakia) had DDD values above 24 in 2014. However, the DDD values were already high for these countries in 2000.

The results show that hospital use of antibiotics, which is poorly reported in the ECDC database, is very low. Eight countries (Latvia, Finland, France, Lithuania, Denmark, Italy, Slovakia, and the UK) had DDD values above 2 in 2014. In the hospital sector, the variability was very high, indeed 3 of the countries (Latvia, Finland, France) started with a higher value, 2 of the countries (Lithuania, UK) had a constant DDD and 3 of the countries (Italy, Slovakia, Denmark) started with a lower value.

The fixed effects regression model confirmed the evidence of this first graphic analysis. Linear and quadratic terms were not significant and these apply to the community (b = 0.07, p = 0.267, 95% -0.06, 0.19, b-squared <0.01, p = 0.813, 95% = -0.01, 0.02) as well as the hospital sector (b = -0.02; p = 0.450; CI 95% = -0.06, 0.03; b-squared <0.01; p = 0.396; CI95% = > -0.01, <0.01).

According to the results, some socioeconomic variables, such as level of education, income, Gini index and unemployment, were not related to the rate of antibiotic consumption both in hospital and in the community (Table 2). Similarly, the variables related to the age and category of prescribing physicians were not related to the rate of antibiotic consumption in the community.

**Table 2:** Results of the regression analysis.

	Antibiotic consumption	Antibiotic consumption
Regressor	in the community	in the hospital sector
	DDD per 1000	DDD per 1000
	inhabitants and per day	inhabitants and per day
Gini index (%)	–0.05	<0.01
	(0.10)	(0.03)
Time effect	2.27	0.93
	(0.006)	(0.528)
*R*^*2*^	0.931	0.745
Countries	22	16
Average obs. per country	12.5	11.1
Mean income of households (€ in thousands)	–0.21	–0.04
	(0.19)	(0.05)
Time effect	1.78	0.66
	(0.044)	(0.805)
*R*^*2*^	0.924	0.672
Countries	20	15
Average obs. per country	12.3	10.9
Educational attainment level (%), 25–64 years,	0.07	–0.02
Upper secondary to Tertiary education (levels 3–8)	(0.08)	0.01
Time effect	1.43	0.68
	(0.138)	(0.791)
*R*^*2*^	0.925	0.673
Countries	22	16
Average obs. per country	14.1	12.5
Unemployment, % of female labor force	–0.05	0.01
	(0.10)	(0.02)
Time effect	2.15	0.91
	(0.001)	(0.549)
*R*^*2*^	0.924	0.664
Countries	22	16
Average obs. per country	14.3	12.5
Total physicians 35–44 years old (% of total physicians)	<0.01	0.08*
	(0.21)	(0.03)
Total physicians 45–54 years old (% of total physicians)	0.13	–0.10*
	(0.18)	(0.05)
Total physicians 55–64 years old (% of total physicians)	0.03	–0.01
	(0.17)	(0.02)
Total physicians 65–74 years old (% of total physicians)	–0.14	–0.05
	(0.25)	(0.05)
Time effect	2.33	2.15
	(0.005)	(0.013)
*R*^*2*^	0.934	0.779
Countries	21	15
Average obs. per country	12.8	10.9
Generalist medical practitioners (% of total physicians)	–0.07	–0.04*
	(0.11)	(0.01)
Specialist medical practitioners (% of total physicians)	0.01	–0.01
	(0.09)	(0.01)
Time effect	2.33	1.49
	(0.005)	(0.125)
*R*^*2*^	0.943	0.796
Countries	22	16
Average obs. per country	10.8	9.4

Notes: Robust standard errors are given in parentheses under the coefficients, and p-values are given in parentheses under the F-statistics of time effect.

The individual coefficient with an asterisk (*) is significant at the 5% level.

The only significant findings relate to the association between the consumption of antibiotics in the hospital and some prescribing physicians’ characteristics, age and category. Our results highlight that an increase of a percentage-point of doctors between 35 and 44 years of age led to an average increase of 0.08 in daily doses per 1000 inhabitants, while a one percent increase in doctors between 45 and 54 years old led to a consumption decrease by an average of 0.10 doses. The results of older age classes (55–64; 65–74) were not significant, but the results confirm the trend observed in the 45-54-year-old group. Additionally, as the percentage of GPs increases, there is an average decrease of 0.04 DDD per 1000 inhabitants in hospitalization consumption of antibiotics.

## Discussion

It is difficult to compare the antibiotic consumption in the community and the hospital sector among the EU countries due the heterogeneous methodologies used to collect data and due to poor availability of data over the years under consideration. Nevertheless, it is clear that there are large variations in community and hospital use of antibiotics in European countries and that the consumption of antibiotics has remained stable over the years. The Netherlands shows the lowest total consumption of antibiotics (11.55 DDD) whereas Belgium, France and Italy had the highest consumption (>30 DDD) in 2014. There was a three-fold difference between countries with the highest and the lowest use of antibiotics. This difference was already present in 2000.

This variability among countries may be affected by antimicrobial stewardship programmes performed in the different European countries. Netherland, which has the lowest levels of antibiotic use, was one of the first countries to adopt a universal, national strategy for AMS in all settings. The Netherland programme started by a Working Party on Antibiotic Policies founded in 1996, supported by the government since 1999. It aimed to provide educational tools in the form of national antibiotic use tests, and in the publication of a national blueprint for an antibiotic policy that can help fellow physicians and pharmacists involved in making such policies at the local level in hospitals and other stations where antibiotics are dispensed [15].

Italy, instead, adopted its first National Action Plan on Antimicrobial Resistance (PNCAR) 2017–2020 in November 2017. The PNCAR represents the tool for implementing the Italian strategy. In order to face the increasing resistance and spread of antibiotic-resistant microorganisms, the PNCAR provides for national coordination, specific objectives and actions through the synergy between national, regional and local levels, the different key stakeholders involved and a governance, in which the roles of the institutions are clearly defined [16].

In Belgium, the Belgian Antibiotic Policy Coordination Committee (BAPCOC) was officially established in 1999 by royal decree. The overall objective of BAPCOC was to promote judicious use of antibiotics and to promote infection control and hospital hygiene, reducing antibiotic resistance and optimizing care. To address these specific tasks BAPCOC founded five multidisciplinary working groups, wich included ambulatory care, hospital care, awareness campaigns, infection control. Specifically, BAPCOC focused on hospital setting through funding of dedicated staff and technical support to antibiotic stewardship (ABS) teams in all Belgian hospitals, the training of over 500 healthcare professionals in ABS, integration of surveillance programs on antibiotic use in hospital [17,18].

It is not easy to explain the reason for high level of antibiotic use in France. Over the last decade, France implemented three national plans to reduce antibiotic prescriptions. As part of the plans, the French government initiated a long-term nationwide campaign to reduce antibiotic overuse and control the dissemination of resistant bacteria in the community. The national program, named “Keep Antibiotics Working,” was launched in 2001, targeting both the general public and health care professionals, to encourage surveillance of antibiotic use and resistance and to promote better-targeted antibiotic use. Since 2002, a public service campaign is launched each winter with the primary goal of decreasing prescriptions. Unfortunately, the impact of these policies has levelled of the antibiotic use, illustrating again the need for an effective antimicrobial stewardship [19].

Antibiotics consumption in the community continues to account for between 85% and 95% of total consumption in 2014. The highest use in the community was in Belgium, France, and Italy (≥28 DDD9) and in the hospital sector in Finland and the UK. The Netherlands had the lowest use in both of the settings (10.6 DDD and 0.9 DDD, respectively).

These findings of substantial stability may explain the behavior of patients and doctors as underlined by the extant literature. A recent review [20] concluded that the patient's demand and the need to give quick relief to their symptoms seem to favor antibiotics prescriptions as doctors, especially those in the community, respond to patients’ needs by prescribing antibiotics rather than providing explanations of why antibiotics may not be needed. In addition, previous studies suggest that some doctors prescribe because they think that patients expect to have antibiotics even when the specific case does not require it [21,22]. To be sure, much of the literature is unanimous on the idea that practitioners’ judgment of patients’ expectations is a major influence on prescribing patterns of antibiotics [23].

According to the results, some socioeconomic variables, such as level of education, income, Gini index and unemployment, are not related to antibiotics consumption in both settings. However, some other extrapolations may be made. Although the impact is not significant, it seems that increasing inequality (Gini index) and unemployment rates reduce the consumption of antibiotics in the community. There are no studies that investigate the correlation between income inequality, unemployment and consumption of antibiotics. A study conducted in Switzerland reported a weak association between income inequality and antimicrobial consumption, but no causal link has been established [24]. To justify this correlation, mediating factors are needed, such as the unequal access to health services. Inequality in income distribution and poverty can considerably reduce access to health services even if they are available [25]. Another factor is inequity in the clinical consultation and the prescribing physician favoring patients who are financially better-off in several of the OECD countries [26].

The results also show that increasing the average income reduces antibiotic consumption. Filippini’s study on socioeconomic determinants of regional differences in outpatient antibiotic consumption supports this finding, highlighting the notion that income is negatively related to antibiotics use [7]. In addition, the importance of wealth has been reported again and again by the WHO [27,28]. In this regard, the WHO believes that the likelihood of doctors receiving continuing medical education increases with national income, with high-income countries being more likely to implement policies regarding rational antibiotics use.

Another important finding in this study is that an increase in the proportion of young doctors (<45 years old) may lead to a significant increase in antibiotics prescription and consumption, while an increase in the percentage of older physicians (45–40 years old especially but still > 50 years old) reduces the consumption of antibiotics.

One possible hypothesis is that practitioners’ experience and professional development activities influence physicians practice, and these factors are responsible for the differences in antibiotics prescribing patterns. This suggestion is supported by some literature [29,30], whereas several qualitative studies have demonstrated that physicians tend to meet patients' antibiotics demands over time instead of educating them properly about rational drug use [23,31].

Another important but unexpected result is that as the number of general practitioner increases, the antibiotics consumption is reduced both in the hospital and community sectors. It is difficult to find an explanation for this phenomenon. A previous study has shown some relationship between ambulatory antibiotic use and hospital use. It highlighted the idea that when the use of antibiotics in one country is high in the community context, it is also likely to be high in the hospital context and vice versa. Assuming that there is a causal link between the percentage of general practitioners and antibiotics consumption in the hospital, the mediating factor that may be most important is the antibiotics consumption pattern in the community.

The reduced use of antibiotic may be attributed to the fact that by increasing the number of general practitioners in the area, they are able to visit patients, establish a relationship of trust and prescribe antibiotic therapy more appropriately.

The main weakness of this study is that the analysis of the trend of use was conducted for all antimicrobials without studying the trajectory of individual antibiotic groups. A focus on individual typologies would help to better understand what may justify the stability of the trend and the typologies with a divergent behavior. A second weakness lies in the fact that the epidemiology of the population of the European areas has not been analyzed. A study of the prevalent pathologies would allow a further explanation of this trend in antibiotics use.

Using different indicators of socioeconomic status and different categories of prescribing physicians and insights from extant literature [32,33], this study’s use of a variety of possible predictors tends to be its strength. Our analyses contribute to the debate on the pattern of prescription and use of antibiotics. This study provides some understanding about relevant determinant and suggests that DDDs are significantly shaped by the category and the age-group of prescribing physicians.

Although it is a known fact that antibiotics consumption varies between countries, the current study confirms this link through analyzing a 15-year period and 22 EU countries as well as shows that the consumption of antibiotics has remained stable over the years. These results may promote and help to define more effective health care policies to reduce the inappropriate use of antibiotics.

## Supporting information

S1 Dataset Supporting data. (XLS)
